# Supplementary figures and images for: Dual-source abdominopelvic computed tomography: Comparison of image quality and radiation dose of 80 kVp and 80/150 kVp with tin filter
Source: PLoS One. 2020 Sep 3;15(9):e0231431. doi: 10.1371/journal.pone.0231431 (PMC7470424; doi:10.1371/journal.pone.0231431)

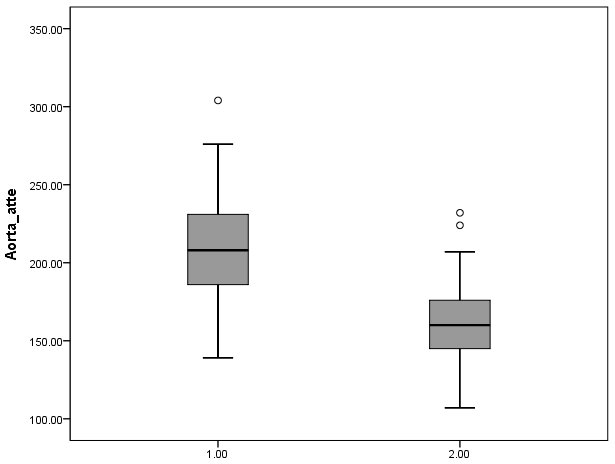

Supplement: S1 Fig — (TIF) [file pone.0231431.s002.tif]

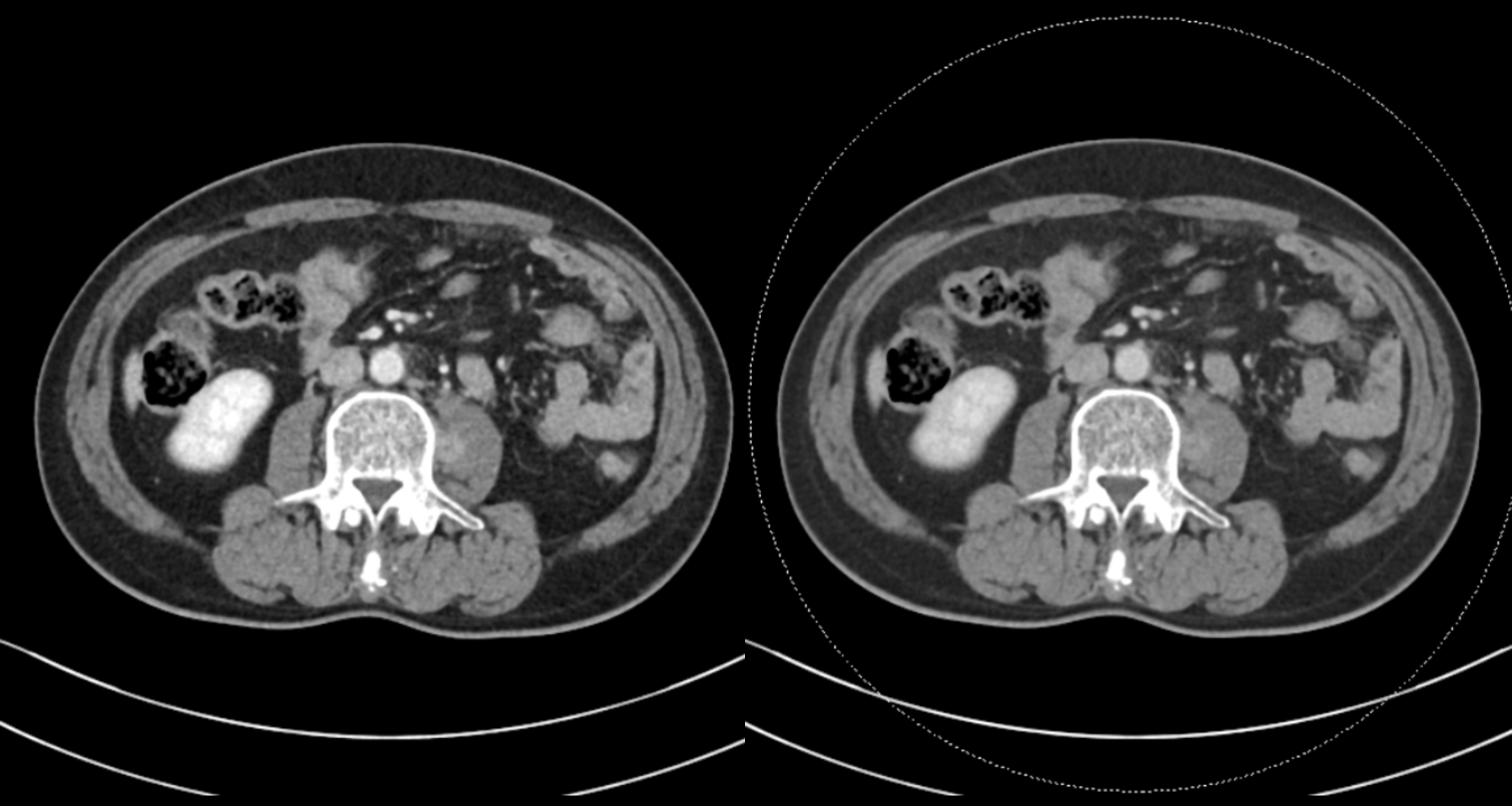

Supplement: S2 Fig — (TIF) [file pone.0231431.s003.tif]
